# Hereditary Predisposition in Phthisis

**Published:** 1907-09-14

**Authors:** J. W. Miller

**Affiliations:** late Assistant Medical Officer of Health, Blackburn


### HEREDITARY PREDISPOSITION IN PHTHISIS.

By J. W. MILLER, M.D., D.P.H., late Assistant Medical Officer of Health, Blackburn.

During the years 1904, 1905, and 1906 I investigated 426 cases of phthisis in Blackburn; of these 144 were notifications and 282 deaths. Inquiries were made in connection with the notified cases only with the consent of the medical attendant. The 282 deaths investigated represented 72 per cent. of the total number of deaths from phthisis which occurred in Blackburn during these three years. Inquiries were made in each case regarding family history of phthisis, source of infection, occupation, previous recent illnesses, social habits, and other factors bearing on the disease. Of the 426 cases investigated, in 116 or 27.2 per cent., there was a family history of phthisis. In 40 of these cases one or more brothers or sisters, but no other members of the family, had died from phthisis or were suffering from the disease. These we shall tabulate as Group II. Group I. contains the other 76 not included in Group II. The following are the particulars of the cases in Group I., of whom 37 were males and 39 females:—

(1.) Relatives affected on mother's side, 29 cases:—Mother alone, 10 cases; mother and sister, 2; mother and brother, 1; mother, uncle, aunt, and cousin, 1; mother, sister, and two cousins, 1; aunts, 2; three aunts and grandfather, 1; two aunts and brother, 1; aunt and uncle, 1; aunt and two uncles, 1; uncle, 3; uncle and brother and sister, 1; three sisters and a brother, 1; grandfather, 2; cousin, 1.

(2.) Relatives affected on father's side, 42 cases:—Father alone, 14 cases; father and brother, 5; father and father's relations, 1; father and uncle, 1; aunt, 6; aunt and cousins, 1; aunt, two uncles, and grandmother, 1; aunt and uncle, 2; aunt and two uncles, 1; uncle, 2; uncle and brother, 1; uncle and sister, 1; grandmother, 2; grandmother and sister, 1; grandmother and brother, 1; relations, 1; great aunt, 1.

(3.) Father and mother's side:—(*a*) Two aunts (mother's side), an aunt (father's side), and two brothers. (*b*) An uncle (mother's side), father and brother. (*c*) Grandmother (father's side) and an aunt (mother's side).

(4.) Unclassified:—(*a*) Cousins; (*b*) uncle and a brother of consumptive. In 37 cases, practically half, the father or mother had suffered from phthisis. In 28 cases, about one-third, uncles or aunts alone had suffered from phthisis. In 65 cases the relationship with the affected person was a close one, either through the father or mother, or brothers or sisters of mother or father. Probable sources of infection were definitely traced in 10 cases.

### Occupation.

Of the 76 cases, 60 had followed an occupation as follows:—Cotton operatives: 18 weavers, 5 winders, 2 spinners, 3 cardroom hands, 1 ironer, 1 reeler, 1 rover; 3 foundrymen, 2 hawkers, 1 commercial traveller, 1 billiard marker, 1 publican, 1 musician (theatre orchestra), 2 cabinet-makers, 1 carter, 1 farm servant, 1 soldier, 1 baker, 1 grocer, 1 bootmaker, 2 tailoresses; 1 sorter of rags in a paper mill, 1 employed in a shoddy mill, 2 paper-bag makers, 1 cotton-reel maker, 1 heel knitter, 1 dressmaker, 1 washerwoman, 1 laundress, 1 cork cutter. Certain of the above occupations would predispose to phthisis—namely, the dusty occupations followed by two spinners, three card-room hands, a sorter of rags in a paper mill, and a male employed in a shoddy mill. Hawkers also suffer a high mortality from phthisis.

Foundrymen suffer a fairly high phthisis death-rate on account of the inhalation of hard metallic particles of dust and also exposure to alternations of temperature.

### Social Habits and Previous Illnesses.

Intemperance is known to predispose to phthisis: in nine of the 76 there was good evidence of alcoholic excess. A foundryman had suffered from pneumonia a short time before the onset of phthisis. Two female weavers had suffered from influenza and bronchitis a short time before the onset of phthisis; both were temperate. A winder had been anæmic after a confinement which occurred a few months previous to the onset of phthisis. A boy aged two years and ten months had suffered from scarlet fever and double otorrhœa a few months before the onset of phthisis.

Of the cases in Group II., 10 were notifications and 30 deaths. There was a possible source of infection from a brother or sister in 17 cases. Here the disease appears to have been transmitted from one person to another, and there may have been no hereditary predisposition.

Thirty-three of the patients followed an occupation, and among them were 20 cotton operatives—13 weavers, 2 overlookers, 3 winders, 1 loomer, 1 spinner. In the other 13 the occupation was as follows:—1 bottler in a brewery, 1 carter, 1 coal-heaver, 1 labourer, 1 horse dealer, 1 caretaker in a school, 1 shunter, 1 schoolmaster, 1 dressmaker, 1 foundryman, 1 tailor, 1 joiner, 1 printer.

Of the above occupations, it is known that coal-heavers, foundrymen, and printers suffer a fairly high death-rate from phthisis. Six of these patients had been intemperate.

### Dwelling Houses and Sanitary Conditions.

The bottler in the brewery had suffered from influenza and bronchitis preceding the onset of phthisis, a winder had had bronchitis for some years, and a spinner diabetes preceding the onset of the disease. A weaver had suffered from anæmia after a confinement a short time previous to the onset. Five of the patients in Groups I. and II. had lived in damp houses; four more inhabited small and badly ventilated rooms in dirty houses; but on the whole the sanitary condition of the houses was good. Most of the houses were four-roomed, and the average number of persons per house was five. In the majority of the cases the only room in which isolation was possible was the front living room, and such isolation, at the best, was imperfect. Paper or rags had been used to collect the expectoration in most cases, and these had been immediately burnt after use.

### Some General Conclusions.

Although there was a family history in 27 per cent. of the cases, in 9 per cent. brothers or sisters alone were affected with the disease. Excluding these latter cases, there was a possible source of infection in 10 of the cases in Group I., and in at least half of the cases there were also other predisposing factors such as occupation, intemperance, recent previous illnesses, etc., some of which were probably more important factors than hereditary predisposition. In Group II., in nearly half the cases direct infection was probably the important factor, rather than hereditary predisposition, whilst occupation and other predisposing factors were also present in about half the cases.

Where investigation has been made regarding family history of phthisis in connection with persons with or without phthisis, it has been found that in a fair percentage of those persons without phthisis there was a family history of that disease. Burckhardt carried out an investigation in 500 families, 250 tuberculous, and 250 non-tuberculous (Zeit f. Tuberk und Heilstatten, Feb. 1904), and found that there was, or had been tuberculosis in some other members of the family in 66 per cent. of the former and in 42 per cent. of the latter. He also found that many more brothers and sisters of the tuberculous than the non-tuberculous had been affected, showing that in a certain percentage direct infection rather than hereditary predisposition was the important factor. The majority of the cases investigated in Blackburn were due to causes other than hereditary predisposition, namely, occupation, and debilitating causes such as previous recent illness, insufficient food, intemperance, and insanitary surroundings.

